# Ethics in scientific research and in the publishing of papers in biomedical journals

**DOI:** 10.1016/S1808-8694(15)30561-9

**Published:** 2015-10-19

**Authors:** Ivan D. Miziara, João Ferreira de Mello Júnior

**Affiliations:** Adjunct Editor - RBORL Associate Professor – University of São Paulo. Full Professor of Forensic Medicine and Medical Deontology - FMABC.; Associate Professor – University of São Paulo.

This discussion about ethical and bioethical issues in scientific research with human beings is very much in need. Most of it because of the 5th article of the Biosafety Law, on the use of embryonic stem cells for research purposes, such fact has stirred not only the scientific community, but also the entire society.

The matter was finally settled by a decision from the Federal Supreme Court, by six votes against five, that there is no legal hindrance to such types of experiment.

For this reason, it is important to retake the theme of Ethics in Research, especially research involving human beings. Society, to whom we ultimately owe explanations, expects and demands from scientists and researchers that they always have in mind the limits that may and should be imposed to their actions and experiments.

The development of sciences in general, especially that of biological sciences, in the last century has been so great that today it is believed we have in the world more scientists than the sum of all of those who have already lived and died.

Based on this astronomic figure only, it is not wrong to suppose that there is and there will be increasingly more knowledge and new technologies produced for mankind.

On the other hand, it is more than obvious that everyday we have more research, especially in health-care, including people as subjects. In other words: research that use humans as their object of study, even when these studies intent to discover new possibilities for cure or treatment of the most diverse disorders.

Scientific research with human beings and for human beings has become a reason of concern in the western world after the discovery and disclosure of the atrocities committed by the Nazis during the 2nd world war, including “scientific” experiments in humans.

Even more frightening is to know that such experiments were carried out or counted on the participation of researchers from the Nazi party, but who were very prestigious in the scientific world of those days; and with the help of research supporting entities and other agencies which had been created to promote health care to the population.

It was only then, after acknowledging the so called “crimes against humanity”, in 1947, that the Nuremberg Code was created, establishing the first norms to regulate research with humans.

Among these rules, there is the need for a voluntary free consent from the subject; for prior studies being carried out in laboratories and with animals; for the analysis of risks and benefits that the investigation may bring about; for the individual's discretion to leave the study at any point during the project and for a proven qualification of the researcher to carry it on, among other issues.

It was already clear in this first (and we could say, already late) regulatory code, one of the cornerstone principles of bioethics, that of the subject's autonomy, or as we said before, the “object-means” of the research.

Even then, abuse continued to happen. In 1996, in the paper Ethics and clinical research, published by the New England Journal of Medicine, Beecker stressed the numerous research projects with human experimentation which were being carried out without proper ethical austerity, and even then published in renowned journals among the scientific community.

In these terms, the 1964 Nuremberg Code revision, during the 18th Assembly of the World Medical Association, became another important landmark in the history of research with human beings. The so called “Helsinki Statement” was unprecedented in presenting the need for an independent committee to review these protocols.

In the following decades, the Standards have been revised and updated, peaking with those aiming at medical research without therapeutic ends, established by the 1996 48th Assembly, in South Africa.

In the same year, in Brazil, a multidisciplinary group created the 196/96 Resolution at the National Health Council, regulating the Standards to be followed when carrying out research with human beings. Among other provisions, which included the periodic review of such standards, the resolution embodied not only the individual's autonomy, but also other important principles of Bioethics, such as doing good and not harm, justice, as well as equality and the right to confidentiality and privacy.

Moreover, it is worth remembering other important provisions of this resolution, such as the concept of risk, which included not only the physical aspects, but also psychological, moral, cultural and social issues. On the other hand, it makes clear that “every procedure (of any nature) which is not fully established in the literature shall be considered as research with human beings”.

Obviously, many important topics were present in this resolution, among others; there is the need for a free informed consent, respect for the participant's vulnerability, the need to justify the use of placebos, demonstration of the preponderance of benefits, and one of the most relevant: the possibility for the “object-means” of the research to have access to the data obtained.

In these regards, the World Health Organization (WHO), as of the XXI century, has spared no efforts to create a universal data base of open access with the ongoing clinical trials involving human beings.

It is within this context, the bounding of all scientific experiments with human beings by well defined ethical principles that the editors of scientific journals (ultimately the means through which these experiments are disclosed) started to get involved with the ethical principles of the research being published.

The rationale is logical: Ethics is not restricted only to the research preparation and the rules for the “object-means” participation, humans. It is completed with its publication in a scientific journal, and the editor of such publications can not help but act ethically, even checking the procedures used by the researchers.

Thus, the International Committee of Medical Journals Editors (ICMJE), in a joint effort with the WHO, established that they would only accept papers with on going clinical trials which had been validated by the WHO and ICMJE, starting in September of 2005.

As far as clinical trials go, the ICMJE defines every prospective research project which subject human beings to intervention and comparative study groups of cause-effect between a medical intervention and its results concerning the individual's health. (http://www.who.int/ictrp/faq/en/index.html).

“Medical intervention” is defined as any procedure that may alter the individual's health, such as the use of drugs and medication, surgical procedures, behavioral therapies, use of equipment, and others.

The Brazilian Journal of Otorhinolaryngology could not refrain from participating in this process and acknowledges the importance and validity of these initiatives by the WHO and the ICMJE.

For these reasons, since 2007, we can only accept for publication in our journal, clinical trial papers that received an ID number in one of the Clinical Trial Registers validated by the criteria established by the WHO and the ICMJE, which web site addresses are available in the ICMJE web site http://www.icmje.org/ in the link “Frequently Asked Questions”.

For the authors, at the end of the paper submission process, we have added a checklist covering many aspects associated with text general structure and format that must be complied with. We reinforce that the ID number from the Clinical Trial Register does not replace the need for research project approval by the Ethics Committee of the institution where the study was carried out. Therefore, it is necessary to include both numbers in the paper. The register number at the end of the summary and the Ethics Committee Approval Protocol Number in the Materials and Methods part of the paper.

This is one more way to show that the Brazilian Otorhinolaryngology and especially Brazilian scientists in general, are in favor of the scientific progress, but all within Ethical Standards and respecting human dignity.

